# Facilitators and barriers to the early implementation of speaking valves in intensive care: a qualitative study using normalisation process theory

**DOI:** 10.1186/s12913-026-16033-5

**Published:** 2026-11-02

**Authors:** Olivia Andersson, Sara Nousiainen, Patricia Hägglund, Mia Hylén, Maria Sehlin, Fanny Silfwerbrand, Anna-Liisa Sutt, Liza Bergström, Markus Saarijärvi

**Affiliations:** 1 Department of Clinical Sciences, Speech and Language Pathology, Umeå University, Umeå, Sweden; 2 Speech and Language Pathology, Karolinska University Hospital, Huddinge, Sweden; 3 Department of Intensive and Perioperative Care, Skåne University Hospital, Malmö, Sweden; 4 Department of Community Medicine and Rehabilitation, Physiotherapy, Umeå University, Umeå, Sweden; 5 Department of Neurology, Skåne University Hospital, Malmö, Sweden; 6 Critical Care Research Group, The Prince Charles Hospital, Brisbane, Australia; 7 Institute of Molecular Bioscience, The University of Queensland, Brisbane, Australia; 8 Speech and Language Therapy, The Royal London Hospital, Barts Health NHS Trust, London, UK; 9 Department of Clinical Sciences Danderyd Hospital, Karolinska Institutet, Stockholm, Sweden; 10 Department of Neurology, Speech and Language Pathology, Danderyd Hospital, Stockholm, Sweden; 11 Department of Cardiology, Danderyd Hospital, Stockholm, Sweden; 12 Department of Neurobiology, Care Sciences and Society, Karolinska Institutet, Stockholm, Sweden; 13 Gothenburg Centre for Person-Centred Care (GPCC), University of Gothenburg, Gothenburg, Sweden

**Keywords:** Implementation science, Interprofessional education, Qualitative research, Respiration, Artificial, Tracheostomy, One-way valves/speaking valves

## Abstract

**Background:**

The utilization of tracheostomy in conjunction with mechanical ventilation significantly impacts various physiological functions, particularly the ability to communicate verbally. Speaking valves, integrated into the ventilator circuit, restore phonation and are recognised internationally as best practice, yet remain underused in Swedish intensive care units (ICUs). Beyond enabling verbal communication, the early use of speaking valves has been associated with improved swallowing, secretion management, and reduced duration of mechanical ventilation. The TRACH INSPIRE project aims to promote their implementation in Swedish ICUs. This study explored facilitators and barriers to the early interprofessional implementation of speaking valves in tracheostomised, mechanically ventilated ICU patients.

**Methods:**

This qualitative study design analysed 19 semi-structured interview data, pre-implementation, with ICU professionals (intensivists, nurses, physiotherapists, and speech-language pathologists) from two participating ICUs in the TRACH INSPIRE project conducted in January-February 2025. Inclusion criteria were that the participants must have participated in the interprofessional competency training for the TRACH INSPIRE project. No formal exclusion criteria were defined. The Data were analysed using deductive content analysis guided by the Normalisation Process Theory (NPT), which examines how new practices are embedded into routine care.

**Results:**

Twelve subcategories were identified that were organized according to the NPT. Facilitators included a shared understanding of the intervention’s value, strong professional commitment, structured implementation processes, managerial support, clearly defined roles, effective interprofessional collaboration, targeted training, and open communication. Barriers centred on organisational constraints such as limited resources, low patient volumes, lack of a comprehensive implementation strategy, and documentation-related challenges.

**Conclusions:**

Early implementation of speaking valves in mechanically ventilated, tracheostomised ICU patients is supported by high interprofessional engagement, shared recognition of benefits, and structured training. However, operational limitations, small eligible patient populations, and documentation burdens present notable challenges. Inclusive team strategies, maintaining competencies, and streamlined processes may promote sustainable integration into routine ICU practice.

**Supplementary Information:**

The online version contains supplementary material available at https://doi.org/10.1186/s12913-026-16033-5.

## Background

In 2025, a total of 18,329 patients required invasive mechanical ventilation (MV) in Swedish intensive care units (ICU), of which 1,021 required a tracheostomy (i.e., 5.57% of all MV cases) [1] Patients admitted to intensive care units (ICUs) often require advanced respiratory support, including MV, which partially or fully assumes the work of breathing [2]. When prolonged ventilation is anticipated, a tracheostomy is typically performed to facilitate respiratory management, being able to wake the patient from deep sedation and reduce complications associated with long-term endotracheal intubation [3]. Tracheostomy involves inserting a tube directly into the trachea through an opening in the anterior neck. Once in place, and with the cuff inflated to seal the airway, no airflow passes through the vocal cords, resulting in loss of voice [4, 5]. Being awake and not being able to communicate due to the tracheostomy, can lead to substantial distress for patients, particularly as this prolonged voicelessness impairs communication and autonomy [6, 7].

Despite these limitations, research has shown that phonation during MV is feasible using speaking valves (SV) integrated into the ventilator circuit (i.e., one-way valves - OWV) [8]. SVs are one-way valves that permit inspiratory airflow through the tracheostomy but redirect expiratory airflow through the upper airway, enabling voice production [4]. Their use requires cuff deflation and careful patient selection but has been associated with multiple benefits including improved lung recruitment, communication, swallowing, secretion management, olfaction, and psychological well-being [9–12].

Despite international best practice guidelines, in Sweden, SVs are currently used almost exclusively in patients no longer dependent on MV [13], and their integration into ventilator circuits is not routine [2]. Knowledge is lacking on reasons for this practice-knowledge gap but may include ICU staff’s lack of knowledge on the use and benefits of SV use. As such, this practice-knowledge gap may deprive tracheostomised ICU patients of the opportunity to regain their voice and benefit from the associated improvements in care and recovery.

The TRACH INSPIRE (TRACHeostomy INterprofessional practice for earlier SPeaking valve Intervention for improved communication, weaning and REhabilitation: implementing Evidence-based practice) project was initiated to support the early use of SVs during ongoing MV in Swedish ICUs. While the physiological and clinical benefits of SVs are increasingly recognised [4, 14, 15], their implementation into routine care remains limited and challenging.

Effective implementation of SVs / OWVs, particularly while the patient is still connected to the ventilator, requires a highly skilled interprofessional team. Patients that are mechanically ventilated via tracheostomy present with complex needs, and successful care depends on coordinated action among physicians, nurses, physiotherapists, and speech-language pathologists (SLPs) [16, 17]. In Sweden, however, the integration of SLPs into ICU teams is not routine, despite their essential role in supporting communication and swallowing rehabilitation [5]. Their introduction into this context represents an important need for interprofessional teamwork and collaborative practice.

To support this change, the TRACH INSPIRE project employs an implementation strategy centred on “superusers”, with representatives from each profession trained in early, interprofessional SV use. These superusers act as internal facilitators and change agents, promoting peer learning and shared ownership while reducing resistance [18]. Such team-based approaches have been shown to improve ICU outcomes [19, 20]. Yet they remain challenging to sustain, as barriers such as unclear professional roles, communication breakdowns, and emotionally demanding environments can undermine collaboration [20, 21].

To address these complexities, implementation science frameworks can be used to guide and evaluate change processes. Normalisation Process Theory (NPT) is one such framework, focusing on the mechanisms by which new practices become embedded in routine care [22]. NPT highlights four interrelated domains—coherence, cognitive participation, collective action, and reflexive monitoring—that shape implementation efforts. In this study, NPT is applied to understand and guide the implementation process from the superusers’ perspective, exploring how contextual, interprofessional, and organisational factors influence the integration of SVs into Swedish ICUs.

## Methods

### Aim

This study explored facilitators and barriers to the early interprofessional implementation of SVs in tracheostomised, mechanically ventilated ICU patients.

### Design

A deductive qualitative design was used following the NPT [22]. The study was part of the TRACH INSPIRE project aiming to implement and evaluate an early interprofessional implementation of SVs in tracheostomised, mechanically ventilated patients in the ICU. The reporting of the study adhered to the Standards for Reporting Qualitative Research (SRQR guideline) [23].

### Interprofessional competency training

Within in the TRACH INSPIRE project an interdisciplinary competency training took place from September to November 2024, including the completion of 10 online modules, each lasting 6–10 min, over a period of six weeks, culminating in a one-day face-to-face workshop. Intensivists, nurses, physiotherapists, and SLPs from two general intensive care units in Sweden (i.e., university hospitals in the north and south of Sweden) were invited to participate. Inclusion criteria for attending the training programme to become a “superuser” within TRACH INSPIRE included a minimum of three years of ICU experience for intensive care nurses and intensivists. Physiotherapists and SLPs were required to have experience working in ICU/with tracheostomy patients, though no minimum years’ experience criteria were applied as the number of allied health personnel (physiotherapists and SLPs) in the ICU setting were limited. Additionally, SLPs were required to be competent in performing and interpreting Flexible Endoscopic Evaluation of Swallowing (FEES).

The hybrid training included evidence-based, pedagogical learning activities such as (1) blended learning with online modules and workshop activities incorporating literature/evidence, protocols and international guidelines, (2) interprofessional-, peer-learning and workshop demonstrations, (3) problem-based learning, and (4) hierarchical simulation using standard cases (one-day workshop).

### Participants

An email invitation for semi-structured interviews was distributed to all staff who had completed the interprofessional competency training described above. No formal exclusion criteria were defined. The goal was to interview all professionals involved in the TRACH INSPIRE project at the two participating units (*n* = 25), or until theoretical saturation was reached [24]. To enhance transferability of findings, a purposive sampling strategy was used, seeking maximum variation in professional background and organisational context [25]. Interview invitations were sent out in three rounds in January 2025 via email. The invitation included information about the aim of the project and clarified that participation was voluntary. If no response was received within one week, a reminder email was sent. If there was still no reply, the person was contacted individually by a member of the TRACH INSPIRE team. Individuals who did not respond despite reminders were considered unreachable. Interviews were scheduled based on participant availability.

### Data collection

Semi-structured interviews were carried out by two of the authors (OA & SN). The interviewers were research students in the Speech-language pathology programme at Umeå University. The authors had no prior relationship with the participants, nor involvement in the TRACH INSPIRE project, to minimise preconceived assumptions. However, the authors had a positive outlook on the project, with the notion that it could improve practice. The authors had no previous experience in qualitative methods. In preparation for the study, they familiarised themselves with the principles of interview-based qualitative research [26].

Prior to conducting the interviews, a semi-structured interview guide (additional file 1) was developed and revised based on feedback from all co-authors. The guide was informed by the four domains of NPT [22]. According to Finch et al. [27], successful implementation requires that all four domains are actively addressed by those involved. Data collection was conducted between January 10th and February 20th, 2025. The interviews were conducted digitally via Microsoft Teams ™ [28] (*n* = 15), or by telephone (*n* = 4) when preferred by the participant. Both options are considered safe and trustworthy alternatives for data collection as they are provided by a secure network of the university institution. All interviews were audio-recorded using Microsoft Teams™, and the built-in transcription function in the platform was also utilised. For telephone interviews, a digital voice recorder was used.

To allow the interviewers to gain familiarity and ensure trustworthiness in the interview guide, two pilot interviews were conducted. These pilot interviews were evaluated in consultation with two co-authors (MS & PH), with no modifications to the interview guide deemed required. Portions of one pilot interview were transcribed separately by both interviewers and then compared and discussed to ensure consistency in transcription style. The pilot interviews were included in the overall dataset. Interviews ranged from 17 to 55 min (mean 30 min). One recording was lost due to technical issues but was partially reconstructed based on contemporaneous notes taken during the interview and was therefore included in the study. After 15 interviews, the interviewers observed signs of theoretical saturation, with recurring themes emerging and few new insights being generated [24]. This was discussed with the co-authors, and it was agreed that already scheduled interviews would proceed but no new interviews would be arranged, resulting in a final sample of 19 participants.

Interviews were transcribed by both interviewers in adjacent to the interviews. Transcripts were pseudonymised by replacing participants’ names with identification codes. Any words or phrases that could potentially identify individuals were removed during the transcription process. Transcripts and recordings were stored securely in a shared folder on the university’s server, accessible only to the interviewers. Once the final transcripts were completed, all audio recordings were permanently deleted.

### Data analysis

Data were analysed by two of the authors (OA & SN) using a deductive content analysis approach [29], guided by the four core domains of NPT [22]. The analysis followed the three phases outlined by Elo and Kyngäs [29]: preparation, organisation, and reporting. In the preparation phase, the transcripts were read repeatedly to gain a comprehensive understanding of the material. Units of meaning related to the research questions were then identified, comprising one or more sentences [29]. These units of meaning were condensed while preserving their core meaning [30]. Both authors conducted the preparation phase individually before comparing and discussing their findings to ensure a shared interpretation. During the organising phase, a categorisation matrix based on NPT’s four domains was developed in accordance with the research questions to identify facilitating and hindering factors for implementation. The units of meaning were then categorised according to this matrix. To increase analytical precision and nuance, subcategories were created within each main category by grouping similar units of meaning. Throughout this phase, the authors regularly revisited the original transcripts to ensure that the categories accurately reflected the data. The analysis was conducted independently by both authors, and the results were subsequently compared and discussed. The content analysis was reviewed and discussed with all co-authors, which led to further discussions and adjustments. This process was repeated until consensus was achieved. An example of the analysis process is presented in Table 1. In the final reporting phase, the analysis process and findings were described in a detailed and transparent manner, aligned with the study’s aim and research questions. The findings were presented based on the content of the categories and interpreted in relation to NPT. To ensure systematic documentation of the analysis, the authors kept ongoing notes in a shared document throughout the process. To enhance transparency and trustworthiness, illustrative quotations were included to clarify findings and support the categorisation. Data analysis was supported using NVivo version 15 [31]. Interviews, along with data analysis were conducted in Swedish. Then, a large-language model was used to translate the coding and results to English. Translations were then checked and corrected if needed.

**Table 1:** Example of data analysis

Unit of meaning	Condensed meaning unit	Subcategory	Domain
I believe that the more successes we have with the speaking valve, the more resistance will decrease.	Resistance decreases as successes increase.	Experience and success reduce resistance and promote sustainability	Reflexive monitoring

## Results

**Fig. 1:**
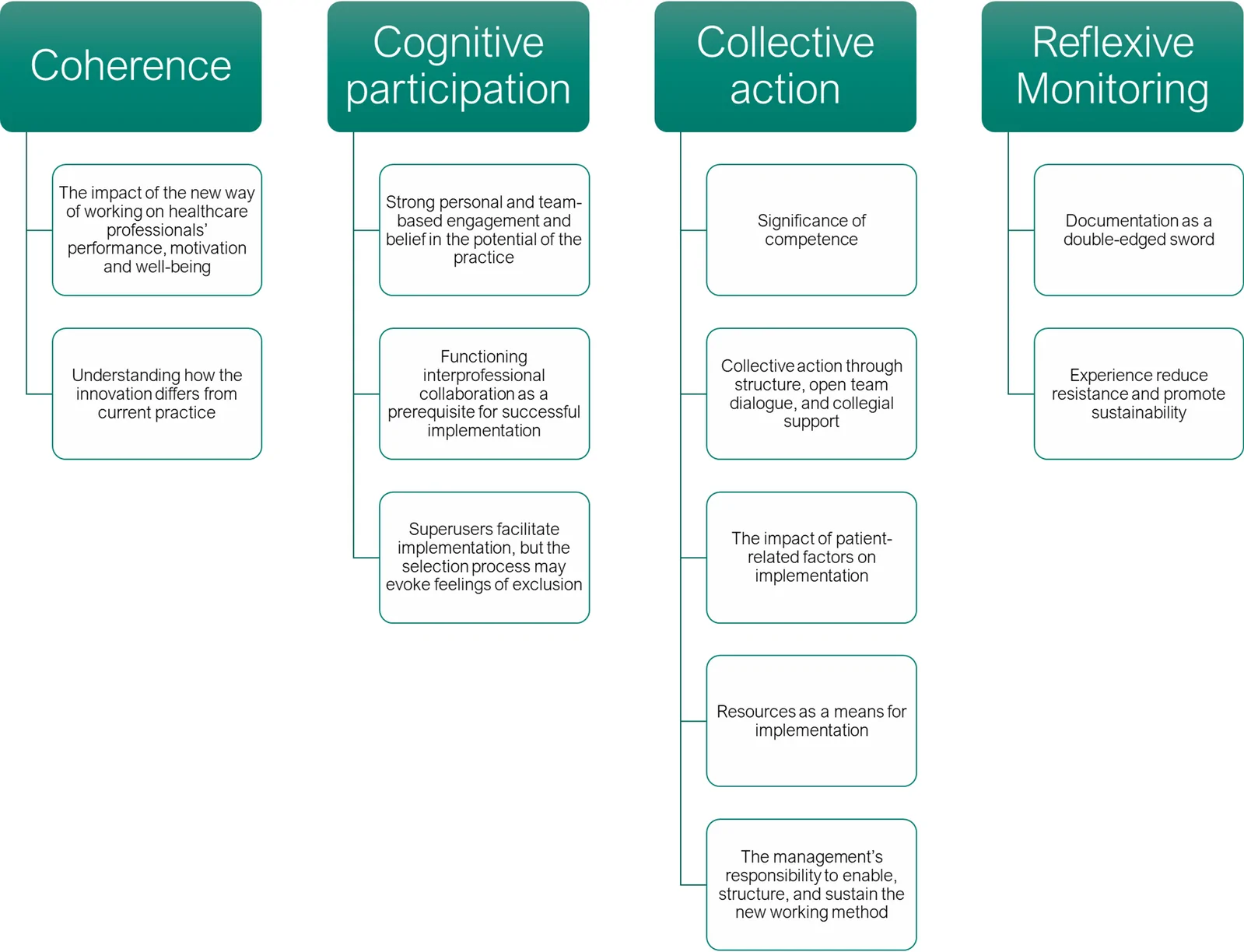
Overview of subcategories structured according to the four domains of NPT (Murray et al. 2010)

Nineteen participants were included, and their characteristics are described in Table 2. The experience of working with ventilator-dependent patients ranged from 2.5 to 32 years (mean 11). The analysis identified twelve subcategories distributed across the four domains of NPT (Fig. 1). These four NPT domains and subcategories are detailed in the text below, accompanied by descriptions and quotes from the participants.

**Table 2:** Participant characteristics

Profession	Participating site *n* (%)		
	ICU 1	ICU 2	Total *n* (%)
Physiotherapist	1 (5.26)	1 (5.26)	2 (10.53)
Speech-language pathologist	4 (21.05)	1 (5.26)	5 (26.32)
ICU nurse	4 (21.05)	2 (10.53)	6 (31.58)
Intensivist	2 (10.53)	4 (21.05)	6 (31.58)
**Total**	**11 (57.89)**	**8 (42.11)**	**19 (100)**

ICU = Intensive Care Unit

Figure 1. Overview of subcategories structured according to the four domains of NPT [22].

### NPT Domain: coherence

Coherence refers to how the new practice is understood and made sense of by those involved, influencing whether it is perceived as logical and meaningful within its specific context [22]. Within this domain, two subcategories were identified, reflecting factors that either promoted or hindered the implementation process (Table 3).

**Table 3:** Identified facilitators and barriers within each domain of NPT and related subcategories

	Facilitators	Barriers
**Coherence**		
*The impact of the new way of working on healthcare professionals’ performance*,* motivation and well-being*	Anticipated improvements in performance Anticipated increase in motivation Anticipated improvements in ICU staff well-being	Perceived risk of heightened stress related to limited time resources
*Understanding how the innovation differs from current practice*	Understanding of the anticipated effects of the new practice Need for consensus on the purpose of the practice	
**Cognitive participation**		
*Strong personal and team-based engagement and belief in the potential of the practice*	Presence of driving team members Personal engagement and belief in the practice Team-based engagement and belief in the practice	Concern that resistance to change may hinder the implementation of the new practice
*Functioning interprofessional collaboration as a prerequisite for successful implementation*	The working approach strengthens interprofessional learning and collaboration Hope for increased interprofessional learning and collaboration	Lack of collaboration may reduce engagement Interprofessional collaboration challenged by diverging perspectives
*Superusers facilitate implementation*,* but the selection process may evoke feelings of exclusion*	The use of superusers is important for driving the implementation	The use of superusers may lead to feelings of exclusion among other staff
**Collective action**		
*Significance of competence*	Tasks are allocated according to professional competence The competence training was comprehensive Need for adequate and continuous training	The division of responsibilities is unclear Excessive staff presence during initial trials A long delay between training and practical application Assistant nurses are not included in the implementation process
*Collective action through structure*,* open team dialogue*,* and collegial support*	The working approach is structured Need for structure Need for open dialogue Need for collegial support	Concerns about the implementation affecting team dynamics
*The impact of patient-related factors on implementation*		There is a limited number of tracheostomised patients Risk that an insufficient number of patients fulfil the eligibility criteria for trial use
*Resources in terms of equipment*,* staffing*,* time*,* and funding*	Sufficient equipment is available Need for adequate staff resources Good planning and foresight facilitate the coordination of all professional groups	Time constraints may hinder implementation Shortages exist in certain professional groups Difficulties coordinating all professions
*Management’s responsibility to enable*,* structure*,* and sustain the new working method*	Management provides the necessary support Need for support from management	
**Reflexive monitoring**		
*Documentation as a double-edged sword*	The documents provide a sense of security and structure	The documents are too long and cumbersome
*Experience reduce resistance and promote sustainability*	Expectation of reduced resistance and enhanced sustainability through experience and success	Risk that unsuccessful trials may trigger a negative spiral

Note: Subcategories in italics

#### Subcategory 1 - The impact of the new way of working on healthcare professionals’ performance, motivation and well-being

Participants provided a varied account of how the new way of working affected their performance. Some perceived that it could enhance performance by increasing knowledge, improving patient outcomes, and promoting identification of patients in need of SVs. Some believed that their performance would remain unaffected, either because performance within their ICU was already considered high or because the new approach constituted only a minor part of daily work. Several participants described the new way of working as motivating, both because the experience of change itself was perceived as stimulating and because the innovation was seen to enhance communication with patients and improve the quality of care.

Participants experience of well-being in relation to the new way of working also varied. Some reported increased well-being because of access to a new and structured way of working, while others highlighted improved opportunities to create value for patients and enhance care delivery as contributing factors. Some participants expressed that their well-being remained unaffected, as their job satisfaction was already high, and the new approach applied to a limited patient group. A few noted that the new way of working could initially be time-consuming and thereby contribute to increased stress. Nonetheless, a recognition of its long-term value was also expressed, which in turn motivated continued use.

#### Subcategory 2 - Understanding how the innovation differs from current practice

Participants demonstrated a broad understanding of how the new way of working could contribute to improvements for the patient, care, and organisation. Some participants highlighted the importance of the entire team working towards a shared goal, as well as the need for consensus among staff and management regarding the purpose. In addition, several emphasised that clearly communicating the innovations benefits could promote shared understanding and increase motivation to apply it in clinical practice.

When asked about the value of the innovation for the patient, participants provided several concrete examples of its positive effects, for example in relation to pharyngeal and airway functions. They described improvements in breathing and swallowing function, coughing ability, and sense of smell, as well as more effective secretion clearance. A recurring theme was that the innovation facilitated voice and speech production. This improved ability to communicate was considered to enhance patient participation in care and to reduce frustration for both patients and healthcare professionals, thereby supporting person-centred care:

For the team, I think it will become easier to care for the patient because the patient will be able to communicate what is working, what is not, and what could be done differently to make it feel better. It will be easier to support the patient in the right way, and you’ll also find it easier to explain why we’re doing certain things. You get a better dialogue with the patient. – Informant 1

Participants also described additional clinical benefits, such as maintaining core stability, which, together with improved communication, could support mobilisation. Additionally, they highlighted the possibility of earlier detection and reduced incidence of delirium. Several participants suggested that the innovation enhancing patient well-being and improved quality of life by reducing anxiety, stress, sleep difficulties, and negative memories from the ICU.

When asked about the potential organisational value of the new working method, participants identified several benefits. These included increased awareness and competence in the care of tracheostomised patients, which may lead to improved quality of care for a vulnerable group. This, in combination with the working method itself, was expected to result in healthier patients, shorter hospital stays, freed-up resources, and financial savings for the organisation. The potential chain reaction, in which better care outcomes lead to further positive organisational effects, was described by one informant:

So overall, I think we might see shorter hospital stays and healthier patients. And perhaps even improved finances if we’re seeing fewer days of care and fewer patients. – Informant 2

### NPT domain: Cognitive participation

Cognitive participation refers to staff engagement and their willingness to actively contribute to the implementation of a new way of working [22]. Within this domain, three subcategories were identified (Table 3).

#### Subcategory 3 - Strong personal and team-based engagement and belief in the potential of the practice

Driving and dedicated team members were highlighted as crucial for enabling implementation, and several participants reported that such individuals were already present within their organisations. At the same time, concerns were raised about resistance to change as a potential barrier. This resistance was attributed to individual attitudes, hesitation towards adopting new practices, or perceived difficulties or low self-efficacy in integrating new ways of working into daily routines. Nevertheless, participants generally expressed positive attitudes towards the new practice, both at the individual and team level:

I feel the team really… the whole team seemed to be in agreement that this is something they want to do, that we see the value in it and that we want to start as soon as possible. – Informant 3

There was a strong sense of engagement and a collective belief that the practice is sustainable, long-awaited, and beneficial for the patient, the team, and the healthcare system.

#### Subcategory 4 - Functioning interprofessional collaboration as a prerequisite for successful implementation

Interprofessional collaboration was considered to support both implementation and long-term sustainability of the new way of working by integrating the expertise of different professions. Participants expressed both hope that the way of working would strengthen interprofessional learning and collaboration, as well as views that it had already contributed to this, particularly through increased collaboration with SLPs. Several expressed appreciations for the presence of SLPs, exemplified by Informant 6, who described them as *“an incredibly welcome and valued addition to our team”*. In addition, expectations were expressed that closer contact with SLPs could promote closer collaboration and enable mutual learning, which may in turn improve the quality of care.

At the same time, it emerged that if interprofessional collaboration was lacking, this may reduce engagement with the new way of working, making well-functioning teamwork a prerequisite. Interprofessional collaboration could also involve varying perspectives and priorities in patient care, which in some cases can create challenges in collaboration.

#### Subcategory 5 -superusers facilitate implementation, but the selection process may evoke feelings of exclusion

Superusers, the selected ICU staff who had undergone specialised training within the TRACH INSPIRE project, form the core group initially trialling the new way of working. Although the general perception was that superusers were facilitators for implementation, concerns were expressed that relying on a designated core group as an implementation strategy may lead to feelings of exclusion and jealousy. One participant described how staff outside of the core group might feel less confident in using the new approach and perceiving this as a form of exclusion:

There are a few of us who are superusers right now. Of course, there might always be someone wondering why they weren’t included in the superuser team. That’s a risk you take whenever you select a few people to do something, others may feel overlooked. That can be a disadvantage. – Informant 6

### NPT domain: Collective action

Collective action refers to the coordinated work carried out by staff to practically implement and integrate the innovation into daily practice [22]. Although no full-scale implementation, the participants described several factors that influence the potential to achieve collective action in five subcategories (Table 3).

#### Subcategory 6 - Significance of competence

Most participants expressed positive views on the competence training completed as part of the TRACH INSPIRE project. The combined theoretical and practical format was highly appreciated. Many highlighted the theoretical component preceding the simulation workshop as particularly valuable, ensuring that all participants shared a common foundational knowledge. While the competence training itself did not present a barrier to implementation, participants suggested that certain elements could have been structured differently to better support the adoption of the new practice, for instance distributing the training over time instead of at one point in time. Most described the training content as well grounded in evidence, while others wanted explicit linkage to research and clinical experience. Additionally, some reported a desire for a more in-depth review of the project’s accompanying case report form (CRF). Conversely, another participant observed that the training placed too much focus on formal procedural aspects and insufficient emphasis on patient care. Despite these varied viewpoints, the interviews revealed an overall positive attitude towards the competence training, thereby supporting implementation.

Although the competence training was generally perceived as sufficient for enabling implementation, several participants voiced concern about the extended interval between training and practical application. This was seen as a risk for knowledge decay, creating a need for repetition and demanding greater precision during implementation. Some participants indicated that a refresher session would be valuable. However, the majority emphasised the need for adequate and ongoing training beyond initial implementation. This was considered essential to reduce the risk of errors and harm to patients. Continued training was also seen as important to ensure that all relevant professional groups, including physicians who do not routinely work in intensive care, possess the necessary knowledge to implement the new practice. It was deemed unsustainable for only a few individuals to maintain competence in conducting trials, as such knowledge is vulnerable to loss through staff turnover or infrequent use. Therefore, the need for sustained competence maintenance efforts across the staff cohort was highlighted.

Furthermore, participants expressed concerns of how tasks were allocated. Most reported confidence in carrying out these tasks and felt that roles were assigned appropriately according to competence. Some remarks were made on the high number of staff involved during trial exercises, complicating coordination and workflow. Another issue concerned ambiguity in role responsibility during the new practice, particularly in situations where multiple professions had overlapping tasks:

Well, it’s perhaps a bit like this at the start that we have to sort out who is responsible for which parts (…) and there could be a risk that the patient is assessed twice or that both parties work on the patient, or that both think ‘this is physio or this is speech therapy’, so that there isn’t a clear agreement. It might be hard to determine exactly who is responsible for what. – Informant 3

Moreover, assistant nurses were not involved in the initial phase of the implementation process, yet their importance was emphasised. Their close contact with patients and broad knowledge were cited as central reasons why this group should have been included from the outset.

#### Subcategory 7 - Collective action through structure, open team dialogue, and collegial support

Collegial support and open dialogue within the team were considered essential prerequisites for achieving collective action and sustainable implementation. A structured approach was emphasised as a foundational condition, ensuring that the new working method could be applied consistently, regardless of which staff members were on duty. Structure was also seen as a prerequisite for offering equitable training to other ICU staff. In addition, participants underscored the importance of open communication within the team, where all members feel safe to express concerns and feedback. Transparent dialogue was described as critical, particularly in situations where the new practice did not function optimally. The need for a clearly defined division of responsibilities ahead of trialling the new approach was identified as a concrete requirement. One participant emphasised that a lack of effective communication could constitute one of the greatest barriers.

Although open dialogue and positive collegial relationships were seen as vital, some reported that the implementation process itself may strain team dynamics. The need for colleagues to support and motivate each other was emphasised to prevent implementation efforts from becoming an additional burden. At the same time, work relationships were described as potentially being negatively affected by factors such as questioning of a profession’s relevance to the implementation, perceived competition, stress, or adverse incidents. To address these challenges, the importance of attentiveness and ongoing open dialogue was strongly emphasised.

#### Subcategory 8 - The impact of patient-related factors on implementation

A potential barrier concerned the limited number of tracheostomised patients, with only a small proportion of these patients were considered meeting the criteria for SV trials. This was seen as a risk that the innovation would be applied infrequently, which may reduce staff confidence and gradually diminish practical competence. As Informant 14 put it: “Volume creates proficiency.” To optimise the process, several participants emphasised the need for tracheostomy to be considered at an earlier stage of care.

#### Subcategory 9 - Resources as a means for implementation

The right equipment and physical facilities were raised as pivotal. While equipment was generally perceived as sufficient, larger and more appropriate facilities to optimise the work process were highlighted to achieve sustainability over time. Regarding staffing, some raised concerns of ensuring sufficient staffing in the future. Others described an existing shortage in certain professional groups, which was perceived as a challenge to implementation.

Time was highlighted as a potential barrier. Specifically, having time to familiarise with the innovation. Successful implementation was considered relying on colleagues to relieve each other of other duties, which was not always possible. Indeed, most participants stressed the importance of having all professional groups present during SV trials. While this was considered a prerequisite, it was a logistical challenge:

One obstacle could be that on some days, it’s difficult to gather the whole team. That might cause a delay of a day or two before we can get started. (…) I mean, if none of the doctors or nurses are on duty, then it’s simply not possible to carry it out – the thing with the superusers – even if you would have liked to. And it can also happen that the speech-language pathologist who come to us are off sick or away, and then things get pushed back a few days as well. But apart from that, I don’t see any major obstacles – it’s just these human factors that can occur. – Informant 7

There were uncertainties on which task could be carried out in the absence of certain professional groups. Some emphasised that successful implementation required full interprofessional collaboration. Others believed that the trials could still be conducted even if not all professions were represented. To manage this challenge, the importance of careful planning was underlined. Suggestions to handle this could be to reduce each team member’s patient load during trial sessions. Several also stressed the importance of providing early notice about upcoming trials to allow for necessary adjustments in staffing schedules.

Finally, financial resources were also discussed. Participants acknowledged that although there are initial costs associated with implementation, these were not seen as a decisive barrier, as the implementation was conducted within the framework of a research project and was expected to result in long-term benefits.

#### Subcategory 10 - The management’s responsibility to enable, structure, and sustain the new working method

To enable successful implementation, management were considered pivotal in creating the necessary conditions. This included ensuring a clear structure, sufficient patient volume, and adequate resources in terms of staffing and time. In addition to facilitating the initial implementation, the participants stressed the responsibility of management in maintaining and sustaining the new way of working. Several participants viewed it as a managerial responsibility to ensure that all staff receive ongoing information and regular training related to the new method.

### NPT domain: Reflexive monitoring

Reflexive monitoring refers to how staff interpret and evaluate the content and execution of the new working method based on their practical experiences [22]. At the time of data collection, most participants had not yet applied the new working method in routine practice. Their evaluations were therefore primarily based on prior experiences of implementation and on the simulation-workshop and generated two subcategories (Table 3).

#### Subcategory 11 - Documentation as a double-edged sword

The TRACH INSPIRE project provided staff with documents including protocols, checklists, flowcharts, and CRFs to clarify each step of the process. Several participants perceived the documentation as extensive and cumbersome, making the documentation process burdensome, creating time pressure, and hindering the establishment of a functional routine:

To be honest, that’s how it feels at the moment, yes. Just the fact that whenever we’re about to do something in this project, we pull out this several-pages-long protocol that we’re supposed to tick off and check through. And I do understand why—it’s partly to ensure we follow the protocol, because otherwise we have nothing to evaluate or relate to. But at the same time, it feels quite rigid, like ‘oh, now we have to check that box’. It’s a bit like going to the supermarket and being told to follow these aisles, first pick up all the dry goods, then this and that. It becomes really strange. You just think—can’t we just go in and shop? We all know what we’re doing, we’ve all done it. So it’s just that it’s a new routine, and that’s why it feels heavy. And we’re probably quite unused to having to relate to that kind of checklist in this way. – Informant 13

Participants expressed concern that the extent of documentation increased the risk of mistakes. Documents were perceived as difficult to apply in practice, particularly when designed for a different clinical context and not fully aligned with local workflows. Nevertheless, others described the materials as contributing to a sense of safety and structure by clarifying responsibilities:

That [Challenges with the method] is of course something to be evaluated, but I think the checklists were good. Everything was very well arranged so that you go through things beforehand, everyone knows their role, and so on—so that it doesn’t end up with no one knowing who’s doing what. It was quite clearly written, and I think that was good. – Informant 8

Furthermore, participants noted that the documents facilitated SV trials by making the process more user-friendly and served as a useful resource for review and knowledge retention. In contrast to the perception that the documents were overly long and complex, others described them as straightforward and easy to follow.

#### Subcategory 12 - Experience reduce resistance and promote sustainability

Some expressed concerns that negative events associated with the use of the new working method could create uncertainty among staff, potentially triggering a negative cycle in which increasing numbers of staff hesitate to apply the approach. In contrast, clinical experience and positive outcomes were considered to provide a stable foundation for the new method, which was perceived to reduce the risk of resistance and reinforce the long-term sustainability of the implementation.

## Discussion

This study applied NPT as a framework to identify facilitators and barriers to the early interprofessional implementation of SVs in tracheostomised, mechanically ventilated patients in two ICUs in Sweden. The findings are discussed in relation to NPT’s four core domains: coherence, cognitive participation, collective action, and reflexive monitoring.

### Coherence

Participants demonstrated a strong shared understanding of the purpose and distinctiveness of the new way of working, perceiving clear benefits for patients, staff, and the organisation. Awareness of the negative impact of prolonged mechanical ventilation without verbal communication [7, 32–34] reinforced the perceived value of the intervention in reducing distress and improving patient well-being. Staff also recognised its potential to reduce professional frustration in communication-limited situations, consistent with Magnus and Turkington’s [34] findings which pointed out interprofessional teams and alternative communication strategies as possible mitigating strategies.

### Cognitive participation

High coherence appeared to foster strong engagement, with participants expressing confidence in the method and valuing interprofessional collaboration—particularly with speech-language pathologists, as also noted by Wiberg et al. [5]. The superuser strategy received mixed responses: while most saw it as supportive, some feared it could marginalise others. Literature suggests such core groups can enhance engagement by fostering peer learning [18]. Potential risks included poor collaboration, differing perspectives, and low readiness for change, reflecting findings by Xyrichis and Rose [20]. Superusers are typically regular staff members who receive additional training to support the introduction of an innovation or routine in the workplace. In this study, some superusers expressed concerns that their appointment created feelings of exclusion among colleagues. Vaag et al. [35] highlight the importance of recognising superusers as leaders of change—an understanding that should be established before their engagement. Clear role definition and adequate support are essential to encourage proactivity and sustain their commitment.

### Collective action

Participants identified both facilitators and barriers to coordinated implementation. The limited patient base—reduced nationally by ~ 200 tracheostomised patients over the last decade [36, 37]—delayed opportunities for practical application after training. Most considered the training adequate, though some suggested multiple formats to suit learning preferences [38] and emphasised the need for refresher sessions to prevent knowledge attrition [18]. Clear role allocation was generally reported, yet some noted role ambiguity and excessive staffing during trials. Including nurse assistants was suggested to enhance inclusivity, echoing Allen et al.’s [18] recommendation to involve all relevant staff. Effective coordination was linked to clear structures, local adaptation, and open dialogue [18, 21]. While resources were mostly adequate, concerns about time and staffing shortages highlighted the need for continued managerial support. The commitment and engagement of superusers also need to be legitimized by managers. The importance of leadership in implementation processes is well established [39]. In this study, the role of leaders as facilitators of practical issues was particularly emphasized, including the allocation of resources to support the project—an area where superusers expressed the need for managerial assistance.

### Reflexive monitoring

As the new way of working had not yet been applied in practice, reflections were largely based on prior experiences and initial workshops. Past successes were seen as confidence-building and potentially reducing resistance [40]. Documentation was valued for structure but could become a barrier if overly lengthy; concise, clinically relevant checklists were preferred [18, 41]. The evolving nature of implementation suggests ongoing review of processes will be essential.

### Strengths and limitations

A qualitative deductive design using semi-structured interviews was appropriate for identifying implementation barriers and facilitators [42]. Deductive content analysis guided by NPT enabled mapping of current progress despite limited practical implementation. Interviewers’ limited preunderstanding posed challenges but may have supported neutrality. Representation was generally balanced, though physiotherapists and speech-language pathologists were underrepresented even though we had more pragmatic inclusion and exclusion criteria for this group (i.e., no formal requirement of 3 years working experience as for nurses and intensivists). Remote interviews facilitated participation but introduced audio and technical issues, potentially affecting transcription. Positive bias may have occurred, as all participants had received competency training.

Another consideration is that some superusers were interviewed prior to having trialled the interprofessional SV practice in real-life after the interprofessional training, thus having limited experience with the new practice, since the interviews were conducted at the pre- or start-implementation period. Different perspectives and qualitative results may be identified at later time-points and further qualitative research at mid and late implementation is warranted. Finally, the interviewers were novice to qualitative interviews. To ensure trustworthiness, they were supervised by a researcher with extensive experience in qualitative methodology and the data collection process as well as analysis was supervised extensively among the co-authors.

This study extends beyond identifying barriers and facilitators by highlighting important considerations for implementing complex interprofessional interventions. Although participants shared a strong understanding of the intervention’s value and demonstrated high engagement, these favourable conditions did not automatically translate into implementation. Instead, organizational factors, including staffing, time, patient volume, documentation and coordination across professions, limited the integration of the new practice. This reinforces a key principle of Normalization Process Theory: successful implementation depends not only on staff motivation but also on organizational capacity to support change [43].

Unlike interventions targeting a single profession or clinical procedure, TRACH INSPIRE required coordinated changes across professional roles, workflows, communication and leadership. Consequently, many barriers were organizational rather than individual, suggesting that education alone is insufficient to achieve sustainable implementation [44].

Several practical lessons emerged. Competency training should be closely aligned with clinical applications to minimize knowledge decay when patient volumes are low. Superuser strategies require transparent communication to avoid perceptions of exclusion, and documentation should balance research requirements with clinical usability. Furthermore, managerial support should extend beyond endorsement to include protected time, adequate staffing and organizational facilitation.

These findings have informed the ongoing TRACH INSPIRE project by strengthening refresher training, broadening knowledge dissemination beyond the superuser group, clarifying professional responsibilities and refining implementation materials. Future research should explore how implementation processes evolve over time and whether barriers identified during early implementation persist, diminish or are replaced as the intervention becomes embedded in routine care.

Thus, these findings underscore the importance of involving all ICU professional groups early, ensuring adequate time for knowledge retention, peer discussion, and refresher training, particularly when eligible patient numbers are low. The superuser strategy should be communicated clearly to prevent perceptions of exclusion. Documentation tools should be concise and eligibility criteria reviewed to broaden patient inclusion. Finally, a renewed needs assessment after sustained implementation could better inform evaluation of collective action and reflexive monitoring.

## Conclusion

This study identified multiple interrelated factors influencing the early interprofessional implementation of SVs for tracheostomised, mechanically ventilated ICU patients, framed within the domains of NPT. Facilitators—such as shared understanding of the intervention’s value, strong professional commitment, interprofessional collaboration, structured training, and managerial support—were present across all domains, indicating a favourable context for change. Barriers were concentrated in collective action and reflexive monitoring, primarily involving operational constraints, limited patient volumes, role ambiguity, documentation burdens, and risks of knowledge attrition over time.

Addressing these challenges will require strategies that maintain competence through ongoing training, broaden staff inclusion, ensure adequate resources, streamline documentation, and adapt eligibility criteria to increase patient access. Strengthening early communication and clarifying roles may enhance collective engagement and coordination. While these findings are specific to the TRACH INSPIRE project, they offer transferable insights for implementing complex, interprofessional interventions in critical care. Sustained managerial commitment, inclusive team structures, and iterative evaluation processes appear essential to achieving long-term integration of SVs into routine ICU practice.

## Supplementary Information

Below is the link to the electronic supplementary material.

Supplementary Material 1

## Data Availability

The datasets analysed during the current study are available from the corresponding author on reasonable request.

## Abbreviations

CRF: Case Report Form
ICU: Intensive Care Unit
NPT: Normalisation Process Theory
OWV: One-Way Valve
SLP: Speech-Language Pathologist
SV: Speaking Valve
TRACH INSPIRE: TRACHeostomy INterprofessional practice for earlier SPeaking valve Intervention for improved communication, weaning and REhabilitation: implementing Evidence-based practice
