# Supplementary material for: Facilitators and barriers to the early implementation of speaking valves in intensive care: a qualitative study using normalisation process theory
Source: BMC Health Serv Res. 2026 Nov 2;26:1512. doi: 10.1186/s12913-026-16033-5 (PMC13729461; doi:10.1186/s12913-026-16033-5)
Supplement: Supplementary file 1 [file 12913_2026_16033_MOESM1_ESM.docx]

# Interview Guide (additional file 1)

## Introduction

As we described in the email, this interview is part of Phase 2 of the TRACH INSPIRE project (TRACHeostomy INterprofessional practice for earlier SPeaking valve Intervention for improved communication, weaning and Rehabilitation: implementing Evidence-based practice).
The aim of this interview is to understand different opinions and perspectives from ICU professionals regarding the implementation of the new interprofessional approach to using the Passy Muir (PM) speaking valve — including earlier introduction of the valve to support communication, rehabilitation, weaning from mechanical ventilation, and decannulation.
This study seeks to explore benefits, drawbacks, and barriers to implementation in depth; therefore, we value all your reflections and comments, both positive and negative.

The interview will be audio recorded and later transcribed for use in the thesis project. Transcriptions will be pseudonymised, and together with the recordings, stored securely and inaccessible to unauthorised persons. Only the authors will have access to the data. In the thesis, the interviews will be presented so that no individual can be identified. Some anonymised quotes may be included to illustrate the results.

By participating, you consent to your responses being recorded and used in the thesis and within the TRACH INSPIRE project.

Do you have any questions about this?
Do you consent to participate in the study and for the collected material to be used for research purposes?

## Initial questions

• First name

• What is your profession?

• At which hospital do you work?

• How many years of experience do you have working with mechanically ventilated ICU patients with or without tracheostomy?

## PART 1. Questions about the implementation of the PM speaking valve — internal/external hospital, healthcare, or societal factors

### Internal factors

• Q1. Could you begin by describing your understanding of what the PM speaking valve for tracheostomised, mechanically ventilated patients mean in your ICU?

• Q2. How do you view the possibilities of introducing the PM speaking valve early for tracheostomised, mechanically ventilated patients in your ICU?

• Follow-up: What parts of your current routines or workflows would need to change or adapt for this method to integrate smoothly? How might this affect your overall work?

• Follow-up: What do you see as strengths regarding the implementation of this approach in your ICU?

• Follow-up: What challenges or difficulties do you think you and your team might face? How could these be addressed or managed?

• Q3. Based on your experience of implementing other new methods or routines, what has contributed to successful implementation and what has hindered it?

• Follow-up: What has been crucial to get everyone in the team to understand and support the change?

• Follow-up: How could these past experiences help in this context?

### External factors

• Q4. From a broader perspective, what benefits could the early implementation of the PM speaking valve have for the organisation or the hospital?

• Q5. Considering the same broader/external perspective, what barriers or threats do you see to implementing this approach (PM speaking valve use for mechanically ventilated tracheostomised patients)?

## PART 2. Participants’ opinions and attitudes regarding interprofessional (IP) teamwork

• Q6. What advantages do you see in interprofessional (IP) teamwork when it comes to introducing early PM speaking valve use for mechanically ventilated patients? Anything else positive about IP teamwork?

• Q7. What disadvantages, barriers, or challenges do you see in IP teamwork related to introducing the PM speaking valve? Anything else negative?

• Follow-up: In what ways could the new way of working negatively affect working relationships?

• Q8. How do you perceive the distribution of new tasks in relation to the team members’ competencies?

• Follow-up: How do you see your professional role as relevant in this new way of working?

• Q9. How will collaboration with your colleagues change when implementing the new approach?

## PART 3. Participants’ opinions and attitudes regarding acceptance, feasibility, and sustainability

• Q10. How do you think the new way of working (interprofessional team implementation of the PM speaking valve) will affect your motivation, performance, and well-being at work?

• Follow-up: How do you think it will impact your motivation? Are there any risks or benefits you foresee?

• Follow-up: How do you think it will impact your performance? Risks or benefits?

• Follow-up: How do you think it will impact your well-being at work? Risks or benefits?

• Q11. What support or resources would you need to successfully adapt to the new interprofessional way of working?

• Follow-up: What role do you think management, or the organisation has in supporting this approach?

• Follow-up: Do you feel that management or the organisation is currently providing this support?

• Follow-up: What would you like management or the organisation to do to facilitate implementation?

• Q12. How do you perceive the training and education provided on early PM speaking valve use to prepare staff for the new way of working?

• Follow-up: What do you think about the scope and content?

• Follow-up: What more would you have wanted?

• Follow-up: How do you experience the routines for reporting effects or outcomes?

• Q13. How sustainable do you think this new way of working will be in the long term? Will it be easy or difficult to maintain over time? Why?

• Follow-up: What is your view on resources (staff, equipment, time) for sustaining this approach?

• Q14. What value do you see in the new way of working?

• Follow-up: For the team? For the patient? For the organisation?

• Follow-up: Do you think staff/the team agree that the new approach is worth investing in?

• Follow-up: In what ways do staff perceptions of the new approach differ?

## Closing

Do you have anything else you would like to bring up or ask about before we finish the interview?

Thank you for your time and valuable insights!
